# Analyzing Reddit Social Media Content in the United States Related to H5N1: Sentiment and Topic Modeling Study

**DOI:** 10.2196/70746

**Published:** 2025-09-09

**Authors:** Oscar Pang, Zahra Movahedi Nia, Murray Gillies, Doris Leung, Nicola Bragazzi, Itlala Gizo, Jude Dzevela Kong

**Affiliations:** 1 Artificial Intelligence and Mathematical Modeling Lab Dalla Lana School of Public Health University of Toronto Toronto, ON Canada; 2 Department of Computer Science University of Toronto Toronto, ON Canada; 3 Global South Artificial Intelligence for Pandemic and Epidemic Preparedness and Response Network Toronto, ON Canada; 4 Africa-Canada Artificial Intelligence and Data Innovation Consortium Toronto, ON Canada; 5 Department of Mathematics York University Toronto, ON Canada; 6 Canada Animal Health Surveillance System Elora, ON Canada; 7 Department of Food and Drugs University of Parma Parma Italy; 8 World Health Organization Geneva Switzerland; 9 World Organization for Animal Health Rue de Prony France; 10 Department of Mathematics Bahen Centre for Information Technology University of Toronto Toronto, ON Canada; 11 Institute of Health Policy, Management and Evaluation University of Toronto Toronto, ON Canada; 12 Munk School of Global Affairs & Public Policy University of Toronto Toronto, ON Canada

**Keywords:** highly pathogenic avian influenza, Reddit, sentiment analysis, United States, topic modeling

## Abstract

**Background:**

The H5N1 avian influenza A virus represents a serious threat to both animal and human health, with the potential to escalate into a global pandemic. Effective monitoring of social media during H5N1 avian influenza outbreaks could potentially offer critical insights to guide public health strategies. Social media platforms like Reddit, with their diverse and region-specific communities, provide a rich source of data that can reveal collective attitudes, concerns, and behavioral trends in real time.

**Objective:**

This study aims to analyze Reddit comments from state-specific subreddits in the United States from the most recent outbreak period of 2022 to 2024 to (1) assess the sentiments expressed as the H5N1 outbreak progresses; (2) identify predominant topics discussed, particularly those corresponding to negative sentiments; and (3) explore correlations between these sentiments or topics and the severity and spread of the outbreak in respective regions.

**Methods:**

We collected 2152 Reddit comments from 160 subreddits across 11 highly impacted states from February 2022 to July 2024. Outbreak data comprising almost 600 entries were obtained from the US Department of Agriculture database. Sentiment classification was performed using a fine-tuned Bidirectional Encoder Representations From Transformers (BERT) base model, and comments were categorized into 6 emotions: anger, fear, joy, love, sadness, and surprise, with a seventh “neutral” category added for low-confidence classifications. Topic modeling was conducted using BERTopic and latent Dirichlet allocation models. Statistical analyses included calculating correlations between sentiment intensity and outbreak severity levels and applying the Mann-Whitney *U* test to assess differences between sentiment categories.

**Results:**

The findings illustrate that H5N1 unfolded in mostly discrete national waves and that only a subset of states—Minnesota and Iowa—experienced chronic, multiwave exposure, a pattern obscured in national aggregates. Sentiment intensity scoring revealed that although 90% (n=1931) of discourse was negative, emotions differed in how they tracked the epidemic: fear aligned weekly with real-time case counts (*r*=0.11), whereas anger, sadness, and even joy surged 3 weeks after the outbreak (*r*=0.20-0.24 after the lag was considered). When both the 3-week lag and an outlier month in terms of outbreak cases were adjusted for simultaneously, those associations strengthened further (overall *r*=0.223), showing how delayed reactions and anomalous surges can mask true sentiment-epidemiology links if left uncorrected. This defines the window in which risk communicators can pre-empt misinformation and economic anxiety. Topic modeling uncovered recurring themes of concern: avian flu culling, sharp egg-price hikes, and frustration over prolonged biosecurity measures. BERTopic provided more coherent and locally specific topics than latent Dirichlet allocation.

**Conclusions:**

Overall, these results underscore the critical role of social media analysis in understanding public reactions, including prevalent themes and sentiments, and guiding timely, targeted public health interventions during the H5N1 outbreak.

## Introduction

The H5N1 virus, commonly known as avian influenza A, is a highly pathogenic virus that primarily affects birds but has the potential to infect other animals as well. It was first identified in domestic poultry in Asia and has since caused numerous outbreaks worldwide, posing significant risks to both animal and public health. The virus can spread among wild birds, domestic poultry, dairy cows, and occasionally to other mammals, including humans, typically through direct contact with infected birds or contaminated environments. Although human infections are rare, they are generally severe and can lead to high mortality rates [1]. The current general concern with H5N1 is its potential to mutate and cause a global pandemic similar to COVID-19, as it can acquire genes that could facilitate easier transmission among humans.

As of the latest reports until December 2024, the spread of H5N1 has been closely monitored, especially in the United States. Since January 2022, there have been over 125 million reported cases of poultry infection, around 11,000 cases of wild bird infections, and 65 confirmed cases of human infection in the country [2]. The first detection of H5N1 in the United States occurred in January 2022 in wild birds, followed by the first commercial poultry outbreak in February 2022, which affected a turkey flock in Indiana [3]. The persistence of H5N1 across various species and states underscores the importance of ongoing vigilance and public health preparedness to mitigate the risk of a potential pandemic. Government agencies such as the Centers for Disease Control and Prevention and the US Department of Agriculture (USDA) have been monitoring the situation through comprehensive flu surveillance systems and public datasets, detailing individual outbreaks in various locations [2].

Social media platforms are invaluable tools for understanding public opinion, detecting mis- and disinformation, and assessing public health concerns in real time. A few studies have analyzed social media data to examine public sentiment and thematic concerns during avian influenza outbreaks. These studies typically use techniques such as sentiment analysis, which classifies emotional tones in user comments, and topic modeling, often using latent Dirichlet allocation (LDA) to identify recurring themes in discussions. Available research has demonstrated that social media plays a dual role—both as a rapid surveillance tool and as a conduit for misinformation. Analyses of tweets and comments related to highly pathogenic avian influenza outbreaks have shown that a significant proportion of content contains emotionally charged language, with predominant sentiments being fear and anger. Many comments also propagate misleading narratives, such as conspiracy theories framing outbreaks as politically motivated events. This underscores the challenge public health officials face in countering mis- and disinformation while effectively disseminating factual information [4].

Beyond mis- and disinformation monitoring, studies have investigated the feasibility of integrating social media data with traditional epidemiological surveillance. For instance, research has examined the correlation between the volume of avian influenza–related tweets and officially reported cases, finding that while social media can provide early warning signals, its reliability as a sole surveillance method is limited due to noise and unverified content [5,6].

Additionally, public perception studies, including surveys of backyard poultry keepers, highlight significant gaps in knowledge regarding biosecurity measures, further emphasizing the need for targeted digital risk communication strategies [7]. Ultimately, while social media presents new opportunities for public health monitoring, its effective use requires a balanced approach—leveraging advanced data analytics for real-time detection while implementing robust strategies to mitigate misinformation and enhance public trust in scientific communication [8].

This research study builds on insights from these earlier studies, combining data collection, sentiment analysis, and topic modeling while introducing methodological enhancements to refine these approaches. The study incorporates data sourced from various Reddit communities segmented by US states, allowing for a more localized analysis of public sentiments and concerns. This approach provides a granular understanding of regional differences, which is crucial for tailoring public health strategies. For sentiment classification, the study uses the Bidirectional Encoder Representations From Transformers (BERT) model, a state-of-the-art tool that outperforms previous models by capturing nuanced contextual meanings in text. In the realm of topic modeling, this research uses both the traditional LDA method and the newer BERTopic approach. By comparing these methods, the study not only gains deeper insights into the themes discussed in social media comments but also evaluates the efficacy and limitations of each model, contributing to advancements in analyzing public health–related social media data.

## Methods

### Outbreaks Data Collection

The data collection for the H5N1 outbreaks used official records from the USDA’s database [9]. The research focused on US states deemed “highly impacted,” identified by those with over 1 million infected domestic birds. The database provided detailed entries for each detected outbreak from February 2022 to July 2024. Each entry on the database included the date, specific location, state, and total number of infected poultry, which was critical for understanding the geographical and temporal progression of the virus.

To prepare the data for analysis, the information from the database was scraped and processed using the Python Pandas library, ensuring accuracy and consistency. The dataset comprised 580 individual outbreak entries, which were systematically organized by state to facilitate further examination and correlation studies.

### Social Media Data Collection

The data collection for the social media content used the PullPush Reddit application programming interface (API) [10] to gather posts and comments (which is referred to as simply “comments” throughout this paper) related to H5N1 outbreaks from various manually identified subreddits representing the entire state and cities within the state for each state involved in this study. The data, collected from February 2022 to July 2024, targeted subreddits from 14 “highly impacted” states experiencing significant H5N1 outbreaks: California, Colorado, Iowa, Michigan, Minnesota, Ohio, Pennsylvania, Texas, Utah, Washington, Wisconsin, Maryland, Kansas, and South Dakota. These states were selected based on the states that reported over 1 million infected poultry from February 2022 to July 2024, based on data from the USDA database.

The API was used to compile a comprehensive dataset by filtering relevant comments from communities using a manually crafted list of H5N1-related words and phrases. To ensure high quality and relevance, comments were cleaned and processed using Python’s Pandas library. Comments with fewer than 5 words were excluded from further analysis, and data from states with fewer than 50 comments were not considered individually, as they lacked sufficient data for meaningful insights. Ultimately, 11 of 14 states retained their own datasets for state-specific analysis. However, all comments from these states were still included in the comprehensive dataset, which comprises 2152 unique comments from 160 different subreddits.

### Sentiment Classification

To classify the collected Reddit comments into various emotional categories, the state-of-the-art BERT base model, specifically the uncased version from the HuggingFace “transformers” library, was used. Developed by Google, BERT is designed to process text bidirectionally, meaning it can consider the full context of any word by examining the words that come before and after it. This deep contextual understanding allows BERT to excel in tasks like sequence classification, making it ideal for sentiment analysis and emotional categorization [11].

The model was fine-tuned using a comprehensive dataset from Kaggle, named “Emotions,” uploaded by Elgiriyewithana [12]. This dataset contains 393,822 English Twitter (subsequently rebranded X) messages, each labeled into 1 of 6 basic emotions: anger, fear, joy, love, sadness, and surprise. The selection of this dataset was strategic, as Twitter, like Reddit, is a vast social media platform with varied community-driven content, making it ideal for training a model to accurately classify sentiments for social media content. The process included first splitting the data into a training and testing set, with 80% (n=315,058) being used for training and 20% (n=78,764) for testing, and then training it on the training set over 3 epochs with a batch size of 16. After training, the model was able to achieve a 94.2% (n=74,196) accuracy rate on the held-out test set.

The BERT model operates by first taking in a tokenized input created by passing an input text into a predefined tokenizer (BertTokenizer in this case), and then processing these tokens through multiple layers to produce contextualized representations of the text. For each input, the model generates scores corresponding to the 6 predefined labels, indicating that the probability of each label being correct. The label with the highest score is assigned as the sentiment of the comment. However, recognizing that some comments might not strongly align with any of the 6 emotions, a seventh label, “neutral,” was introduced. This label is assigned to comments where the maximum score across all categories is less than 0.5, indicating low confidence in the classification and suggesting a neutral sentiment. After training the model, it was used to classify the Reddit comments based on their sentiments and find the intensity (the score generated by the model) of each sentiment class.

### Topic Modeling

For the topic modeling aspect of this study, 2 models were used and compared: BERTopic and LDA. The BERTopic model was chosen for its use of BERT embeddings to capture semantic nuances in the text, closely aligning with the BERT-based sentiment classification model used in this study. BERTopic excels in identifying dynamic and contextually rich topics from textual data, making it ideal for analyzing social media content where discussions can be diverse and rapidly evolving [13]. On the other hand, LDA was used due to its proven effectiveness in previous research, particularly in similar past studies examining social media sentiments. The main difference between the models is that BERTopic leverages context-aware embeddings, which capture the meanings and relationships between words based on their use in sentences, while LDA focuses purely on the distribution of words across topics and the distribution of topics across documents [14].

To evaluate the effectiveness of the topics generated, the CoherenceModel from Gensim was used to calculate overall coherence for each parameter combination across states [15]. This metric, along with the Jaccard similarity formula—used to measure the similarity between sets of top words in different topics—helped assess the uniqueness and repetition of topics. An ideal model maximizes coherence and minimizes repetition, guiding the fine-tuning of both BERTopic and LDA models.

The process for BERTopic involved comprehensive data preprocessing, including the removal of punctuation, common stop words, and lemmatization using Pandas and Natural Language ToolKit libraries. Text data were converted into numerical representations using the CountVectorizer from scikit-learn, with key parameters like “max_features” and “ngram_range” fine-tuned for optimal performance. The Hierarchical Density-Based Spatial Clustering of Applications with Noise clustering model was crucial in identifying clusters of varying densities, with parameters like “min_cluster_size” and “min_samples” ensuring robust clustering. The resulting topics and subtopics were saved into JSON files categorized by state. Similarly, the LDA process included identical preprocessing and vectorization steps. However, LDA differs by requiring the number of topics, represented by the “n_components” parameter, to be specified prior to model training. The topics generated were also saved in JSON files named by state.

### Statistical Analysis

After classifying each Reddit comment by sentiment, they were grouped accordingly, and an intensity score was calculated by combining the strength of the BERT model’s predictions with the volume of comments associated with each sentiment. These intensity levels were then analyzed visually using box plots, which highlighted the distribution of sentiment intensities across categories. Statistical significance annotations were added using Python’s *statannotations* package. The Mann-Whitney *U* test, a nonparametric test for comparing differences between 2 groups, was applied to calculate *U* values and corresponding *P* values, indicating whether observed differences were statistically significant (*P*<.05).

To examine the correlation between sentiment intensity levels and outbreak severity, sentiment intensity values were first standardized to a scale of 0 to 100. Similarly, weekly outbreak data were scaled to a 0 to 100 range, creating standardized “outbreak levels.” This normalization allowed for a direct comparison between outbreak severity and the previously standardized sentiment intensity levels. The Pearson correlation coefficient (PCC), calculated using the “pearsonr” function from the SciPy library, was used to assess the strength and direction of the relationship between changes in individual sentiment levels and the severity of the outbreak.

### Ethical Considerations

This research was conducted in accordance with established ethical principles for web-based research, relying exclusively on data that are in the public domain of Reddit. The H5N1 outbreak statistics were sourced from the USDA, which is a public government agency that provides open access to these records for research and public information purposes. A primary ethical priority was protecting individual privacy in collecting social media data. All data were gathered from fully public Reddit forums known as subreddits, where users post content with no reasonable expectation of privacy. Crucially, the data collection process was designed to be nonintrusive and to programmatically deidentify all content. No personally identifiable information, such as usernames, user profile information, or IP addresses, was collected or stored. The resulting analytical dataset contained only the textual content of the comments, their timestamps, and the subreddit of origin, rendering the data fully anonymous and severing any link to the original authors, following the ethical principles outlined by the Association of Internet Researchers [16] and the guidelines set forth by the International Chamber of Commerce/European Society for Opinion and Marketing Research International Code [17]. To protect the privacy and dignity of Reddit users, our research focused on broad, population-level trends rather than individual comments. The objective was to analyze collective responses, not to judge personal opinions. We complied fully with Reddit’s terms of service when collecting data via the PullPush API, and the datasets were stored securely, with access limited to the research team to prevent any potential misuse. As this research involves the passive analysis of posts, individual informed consent is waived [18].

## Results

### Outbreak and Sentiment Data

**Figure 1:**
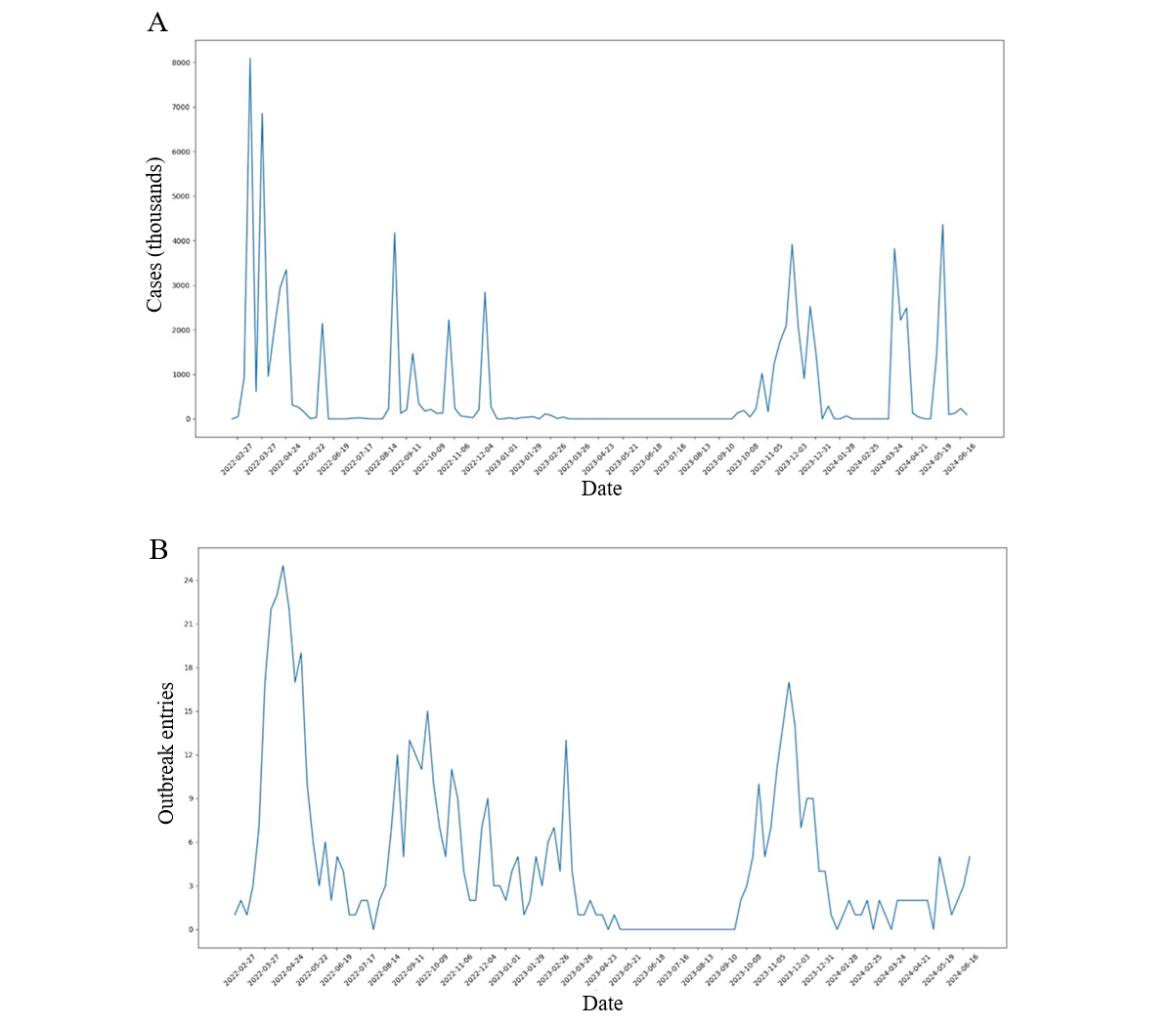
(A) Weekly counts of laboratory-confirmed H5N1 cases in domestic poultry reported to the US Department of Agriculture across 11 highly impacted states (California, Colorado, Iowa, Michigan, Minnesota, Ohio, Pennsylvania, Texas, Utah, Washington, and Wisconsin) from February 2022 through July 2024, illustrating the temporal waves of infection in this retrospective analysis. (B) Weekly number of distinct premises (farms or wild locations) reporting H5N1 outbreaks in the same 11 states and time frame, highlighting the spatial dispersion of infection events.

The outbreak data are illustrated through graphs showing both the number of H5N1 cases reported by week (Figure 1A) and the number of individual H5N1 outbreak entries (locations of recorded occurrences) reported by week in various states (Figure 1B). These graphs provide a detailed comparison of outbreak severity, timing, and distribution across the United States and within the 11 states examined in this study.

When examining the overall graph, it could be broken down into several major spikes in the number of cases and periods with nearly 0 cases. This means that the outbreaks were concentrated in specific weekly time frames with significant surges, followed by intervals of relative inactivity, which suggested that the spread of the virus occurred in waves, with intense periods of transmission followed by periods of containment.

States such as California, Colorado, Iowa, Minnesota, and Ohio experienced multiple weeks with significant spikes in case numbers. This pattern indicates that the outbreaks in these states were more protracted, with multiple periods of heightened severity. These states also tend to have the highest overall case counts. Minnesota, in particular, stands out for having the most evenly distributed outbreak case graph, with multiple spikes across an extended period, far exceeding the other states in terms of distribution. It ranks third in total cases, with approximately 8.4 million cases. Iowa, which recorded the highest number of cases at around 23.3 million, also shows a well-distributed pattern, with consistent peaks over time, reflecting multiple severe outbreaks. In contrast, states such as Michigan, Pennsylvania, Texas, Utah, Washington, and Wisconsin exhibit a different pattern. These states generally experienced a single major spike in cases (over 1 million), with few or no additional smaller peaks.

On the other hand, the number of individual outbreak entries was more evenly distributed across all states. Interestingly, there were no significant differences between states in terms of outbreak entries, with most states following similar patterns. However, Minnesota once again stands out, with 169 outbreak entries, far exceeding the totals for other states, despite it not having the highest total case count. Considering the additional high and well-spread case counts, this makes Minnesota the most severely impacted state when considering multiple metrics. While Iowa has recorded higher total case numbers, Minnesota’s combination of numerous outbreak locations and consistent case spikes over time indicates a broader and more sustained impact of the virus. Overall, these data provide a baseline, against which the public’s sentiment, as expressed in Reddit comments, can be compared.

Using the labeled datasets generated by the BERT model, sentiment classification bar charts were created to display the quantities of comments expressing different emotions for each state. The analysis revealed notable variations between states, but certain trends were consistent. The negative emotions of “sadness,” “anger,” and “fear” emerged as the most dominant, accounting for around 41.9% (n=901), 30% (n=645), and 17.9% (n=385) of comments, respectively. Combined, these 3 emotions represent approximately 90% (n=1931) of all comments, highlighting a clear public tendency toward negative sentiments. Comments categorized under “joy” made up about 7.6% (n=163) of the dataset, reflecting a relatively low level of positive emotional engagement. Comments exhibiting the emotions “surprise” and “love” were scarce, with only 8 comments in total (Table 1). Due to their minimal presence, these emotions were excluded from further correlation analysis with outbreak data. This general sentiment composition was consistently observed across state-specific data. In every state, the number of comments expressing “sadness,” “anger,” and “fear” consistently surpassed those expressing “joy,” maintaining a similar emotional structure regardless of regional differences. While state-specific nuances exist, the overall emotional distribution strongly points to widespread negative sentiment during outbreaks.

When analyzing the comments dataset, the top 10 subreddits of 160 by comment count were identified. Among these, Denver, Seattle, Minnesota, Austin, and Texas subreddits are the top 5, making up around 32.9% (n=708) of all comments (Table 2). This is a very significant amount, considering that there were 80 subreddits with comments scraped. Hence, the number of comments across all subreddits was not evenly distributed. However, if we remove these top 5 subreddits individually from further sentiment analysis, we observe no significant changes in the overall sentiment distributions, with the sentiment rankings by number of comments remaining the same across all cases (Table 3). Thus, these major subreddits do not disproportionately influence the sentiment distribution. Given that the remaining subreddits individually contribute much fewer comments, the fact that removing the top 3 subreddits does not affect sentiment rankings suggests that it is highly unlikely that an individual subreddit, regardless of its comment volume, will substantially impact the overall sentiment analysis. This suggests that the sentiment patterns observed are mostly consistent across various subreddit communities, regardless of their comment volume.

**Figure 2:**
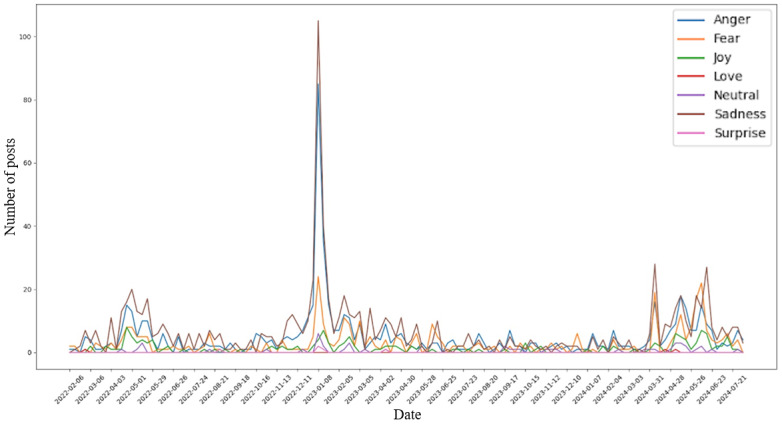
Weekly counts of Reddit comments by emotion category (anger, fear, joy, love, sadness, and surprise) from February 2022 to July 2024, revealing 3 peaks of social media engagement that align with epidemiological waves of H5N1.

To show the labeled sentiment dataset in a similar manner as the outbreak dataset, for each sentiment, the comments that belong to it in the dataset were grouped by weeks and plotted as time series line graphs. An observation can be made here, noting that all sentiments’ changes in quantities seem to follow similar trends in the time period of concern for this study. Furthermore, there seem to be 3 visible groups of spikes in the number of comments for most sentiments across the time period: February 2022 to May 2022, November 2022 to February 2023, and March 2024 to June 2024. These groups, although varying in terms of comment quantities, share the common characteristic of being around 3 to 4 months long before dying down (Figure 2).

**Table 1:** Proportion of 2152 Reddit comments about H5N1 (collected via the PullPush application programming interface from 160 state-specific subreddits in the 11 study states between February 2022 and July 2024) classified into 7 emotion categories (anger, fear, joy, love, sadness, surprise, and neutral) by a fine-tuned Bidirectional Encoder Representations From Transformers model, showing that negative emotions (sadness, anger, and fear) comprise approximately 90% (n=1931) of discourse.

Sentiment type	Posts, n
Sadness	901
Anger	645
Fear	385
Joy	163
Neutral	50
Surprise	5
Love	3

**Table 2:** Top 10 geographic subreddit communities contributing the highest comment volumes to the H5N1 dataset (February 2022 to July 2024), indicating each community’s percentage share of the total 2152 comments.

Subreddit	Posts, n
r/Denver	212
r/Seattle	140
r/Minnesota	128
r/austin	121
r/texas	107
r/Michigan	75
r/LosAngeles	71
r/Sacramento	70
r/Pennsylvania	64
r/SanFrancisco	62

**Table 3:** Comparison of overall sentiment proportions in the full comment sample versus after sequential removal of the 5 largest subreddits, demonstrating that no single community disproportionately influences the observed emotional distribution.

Sentiment type	Posts (overall), n	Posts (minus r/Denver), n	Posts (minus r/Seattle), n	Posts (minus r/Minnesota), n	Posts (minus r/austin), n	Posts (minus r/texas), n
Sadness	901	803	838	849	847	849
Anger	645	568	599	607	612	613
Fear	385	360	366	363	358	369
Joy	163	157	154	153	158	161
Neutral	50	45	47	44	48	45
Surprise	5	4	5	5	5	5
Love	3	3	3	3	3	3

### Sentiment Types and Intensity Scores

**Figure 3:**
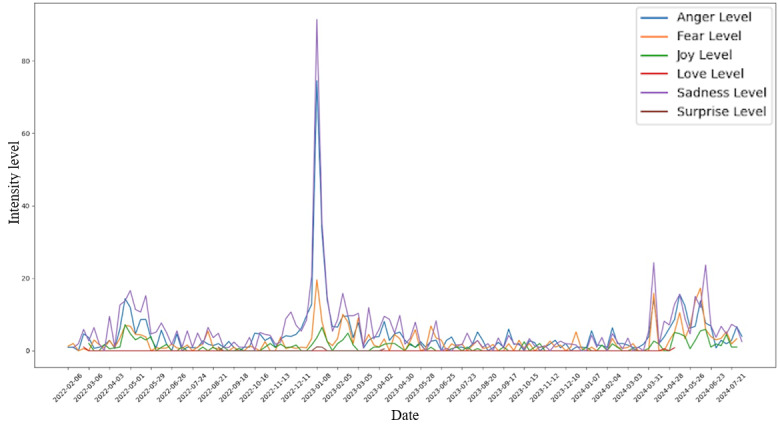
Weekly sentiment intensity scores (number of comments × average Bidirectional Encoder Representations From Transformers confidence) for each of the 6 emotion categories plus neutral, normalized to track weighted public affect over the 29-month study period.

Simply analyzing the frequency of sentiments based on comment counts is insufficient. A higher number of comments reflecting a particular sentiment do not necessarily indicate the intensity of that sentiment. To address this, the concept of “sentiment intensity levels” was introduced, which involves calculating a weighted score for each sentiment per week to represent its overall “intensity” in that week. The calculations leverage the fact that BERT, the sentiment classification model used in this study, generates probabilities for each label, identifying the label with the highest probability as the predicted label. During the classification of the Reddit comments into various sentiments, both the predicted label and its corresponding probability (confidence score) are recorded. Specifically, the sentiment intensity level for a particular sentiment per week is calculated by multiplying the number of comments for that week by the average confidence score produced by the BERT model for the corresponding label within the week. Figure 3 shows the result of applying this transformation to the data.

**Figure 4:**
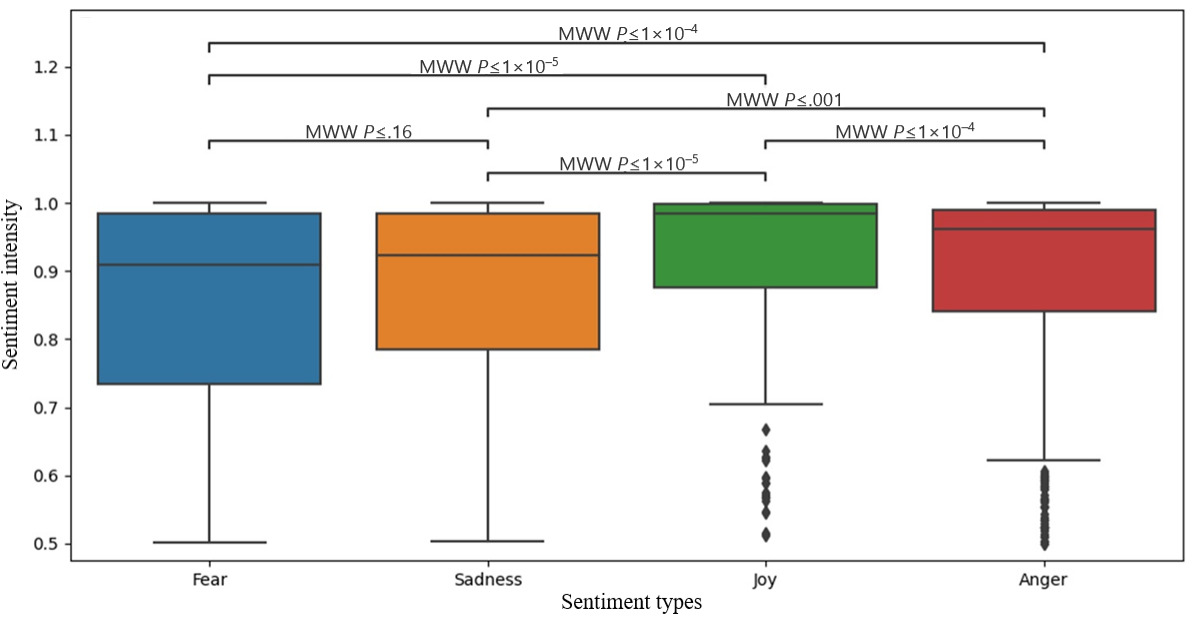
Box-and-whisker plots of weekly sentiment intensity distributions for anger, fear, sadness, and joy (February 2022 to July 2024), with significant differences marked by Mann-Whitney *U* tests (*P*<.05), illustrating that positive sentiment (joy) is significantly lower and less variable than negative emotions. MWW: Mann-Whitney-Wilcoxon.

After applying the Mann-Whitney *U* test [19] to the intensity levels, several pairs of categories contain statistically significant *P* values, with the highest differences being between the “joy” category and all other categories. The lowest difference, which is also represented by the only insignificant *P* value (>.05), is between the “sadness” and “fear” categories. Similar trends are also observed for each state, where “sadness,” “fear,” and “anger” all seem to have differences in distributions that are not extremely significant, while comparing “joy” distributions to the rest, significant differences were exhibited (Figure 4). Other categories were omitted due to insufficient quantities.

### Correlation Between Outbreak Data and Sentiments Expressed

**Figure 5:**
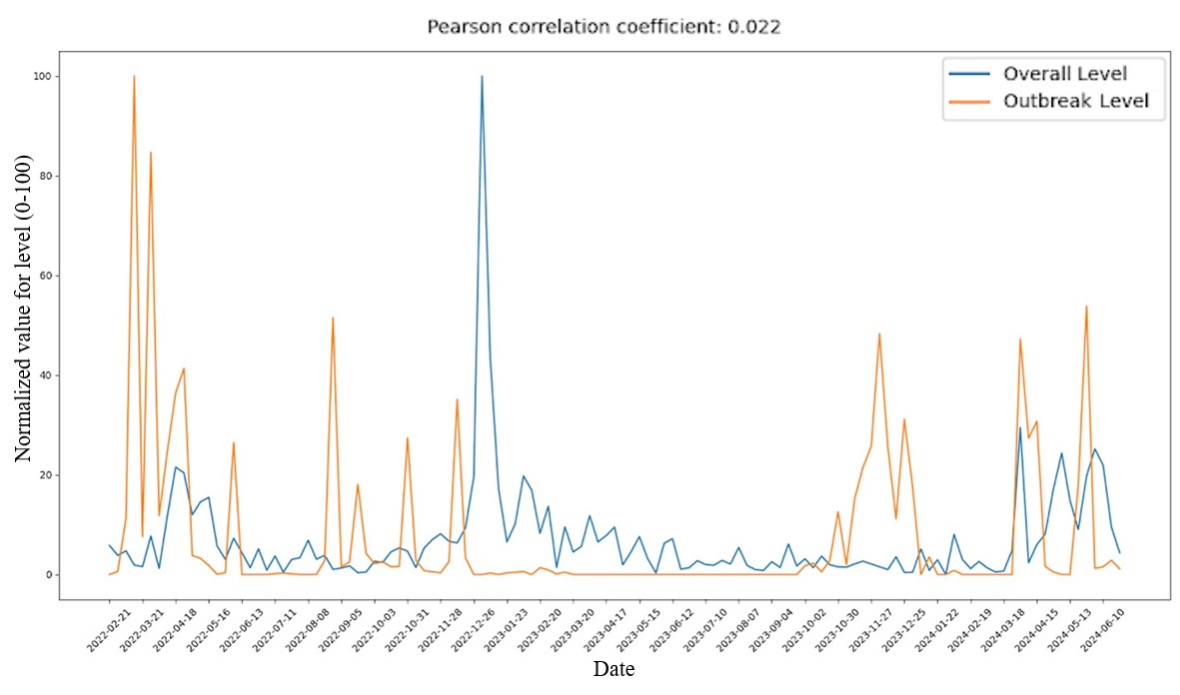
Comparison of aggregate weekly sentiment intensity (sum across emotions, scaled 0-100) versus normalized H5N1 outbreak severity (weekly infected bird counts scaled 0-100) from February 2022 through July 2024, showing minimal real-time correlation (Pearson *r*=0.022) between public affect and outbreak dynamics.

The PCC values were computed using SciPy’s “pearsonr” function to compare the normalized sentiment intensity levels and normalized outbreak severity levels, both scaled to a range of 0 to 100, on a monthly basis. The results indicated no significant correlations, as all PCC values were close to 0. Overall, the PCC of all sentiment intensity levels combined when compared to outbreak severity levels is 0.022, which does not display any significant correlation (Figure 5).

**Figure 6:**
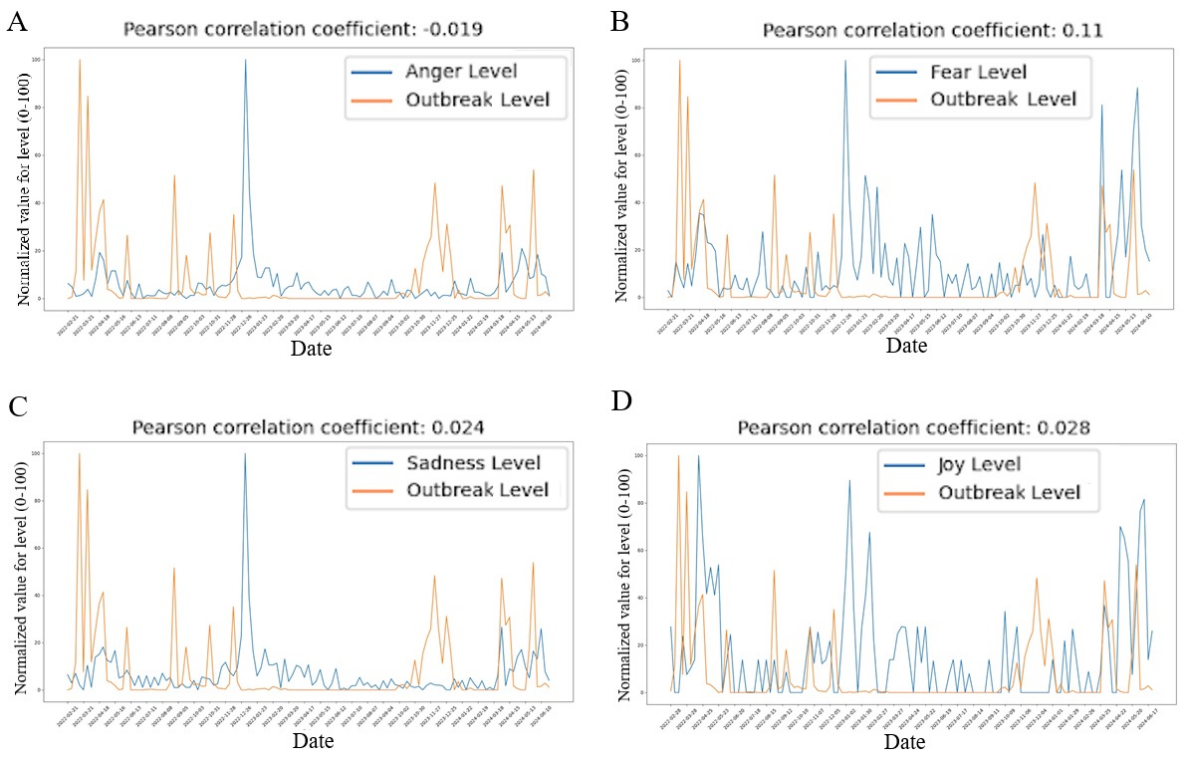
(A) Scatter plot of weekly anger-intensity scores (0-100) versus normalized weekly H5N1 outbreak severity (0-100) across all 11 states from February 2022 to July 2024, indicating a negligible association. (B) Scatter plot of weekly fear-intensity scores versus outbreak severity for the study period, showing a minor real-time correlation (*r*=0.11). (C) Scatter plot of weekly sadness-intensity scores versus outbreak severity, reflecting minimal immediate coupling with case counts. (D) Scatter plot of weekly joy-intensity scores versus outbreak severity, indicating low levels of positive affect with minimal real-time correlation.

When examining the relationship between outbreak levels and various sentiments, a weak positive PCC of 0.11 was found between outbreak levels and “fear.” This was the only significant correlation observed among the sentiments studied. In contrast, negligible correlations were found between outbreak levels and the sentiments of “anger” (*r*=–0.019), “sadness” (*r*=0.024), and “joy” (*r*=0.028). Furthermore, the temporal distribution of “fear” levels across time was relatively consistent, suggesting a more stable association with outbreak levels compared to the other sentiments. The line graph of the “fear” levels by time is also the most evenly distributed across time compared to that of the other sentiments (Figure 6A-D).

**Figure 7:**
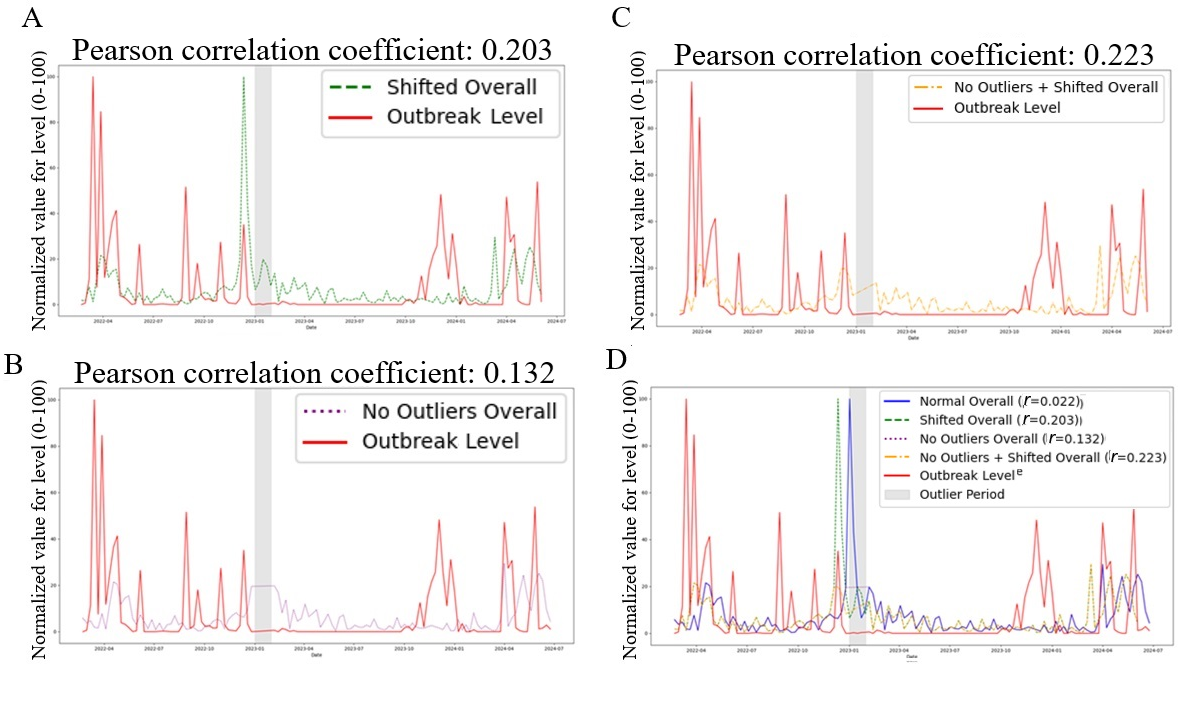
(A) Overlay of aggregate weekly sentiment intensity (sum across emotions, scaled 0-100) versus normalized H5N1 outbreak severity (0-100) across 11 US states from February 2022 through July 2024 after applying a 3-week backward lag to sentiment data; correlation improves to *r*=0.203. (B) Overlay of aggregate weekly sentiment intensity (sum across emotions, scaled 0-100) versus normalized H5N1 outbreak severity (0-100) across 11 US states from February 2022 through July 2024 following removal of January 2023 outlier weeks, yielding stronger correlation (*r*=0.132). (C) Combined lag and outlier adjustment overlay of aggregate sentiment intensity versus outbreak severity for all 11 states (February 2022 to July 2024), achieving the highest correlation (*r*=0.223). (D) Composite overlay of original and all adjusted sentiment intensity series against normalized outbreak severity across 11 US states (February 2022 to July 2024), illustrating how lag correction and outlier control align emotional trends with epidemiological data.

Sentiment trends lagged behind outbreak severity but aligned more closely after adjusting the data. Shifting sentiment scores back 3 weeks and removing January 2023 outliers improved correlations, with PCCs rising across emotions and the overall index (Figure 7A-C). These adjustments revealed that Reddit sentiment mirrored outbreak trends more closely than raw data suggested, though correlations remained moderate. Figure 7D illustrates the stronger alignment after adjustment.

State-specific correlations between overall sentiment intensity and outbreak severity levels were also explored. Although some significant correlations were found between these 2 metrics across several states, most of them occurred in states with a single major spike in the number of cases and having near-zero cases across other weeks, which include Michigan, Pennsylvania, Texas, Utah, Washington, and Wisconsin as identified before. In these cases, the correlation coefficients were not representative of the actual correlations across time. This is because an individual extreme data point can exert a significant influence on the correlation coefficient, potentially overstating the relationship, which is what occurs when analyzing these single-spike states. For the states with multiple spikes over time in cases, the only nonnegligible correlation was a weak positive relationship for Minnesota, with a PCC of 0.231 between overall sentiment intensity and outbreak severity levels in that state.

### Identified Topics

After generating topic clusters for each state using BERTopic and LDA, we organized the results into JSON files. Each file is keyed by topic ID, and each value contains a list of comments assigned to that topic based on probability thresholds. We then used Python’s *wordcloud* package to render each cluster’s most frequent terms—omitting generic words such as “bird,” “avian,” “flu,” “H5N1,” and “influenza” to surface more informative tokens. The resulting visuals showed differences in the top words as well as distributions of the top words across various states, as expected, with the most common words across all states being “people,” “outbreak,” and “egg.” When comparing the 2 sets of word clouds, the BERTopic-derived terms appear slightly more evenly spread across a broader semantic range than the LDA terms, but the gap is modest. Each method surfaces a very different set of top words, but neither cloud clearly outperforms the other in isolating the most prominent discussion themes.

Next, the frequency of each topic across states was analyzed to identify which topics were most common in each state, providing insights into the public opinion on outbreaks. This comparison highlights the topics that are most significant for each state, effectively representing the general concerns of the public regarding the outbreaks.

To do this, the topics identified through the 2 different topic modeling approaches: BERTopic and LDA, were combined into sets that were then saved into JSON files for each corresponding state. The topics identified by the BERTopic method were initially loaded, and then the topics generated by the LDA method were added sequentially, ensuring that each topic had a unique identifier. After combining the topics, each comment within a state’s Reddit comment dataset was matched to the topic it most closely aligned with. This matching was done by first identifying all topics the comment belonged to, using the previously mentioned JSON files, and then calculating the cosine similarity between the text of each comment and the text of each topic. The topic with the highest similarity score to the comment was assigned to the comment, along with the similarity value. When manually examining the topics that appeared the most, and the topics that correspond to comments that exhibited the highest comprehensive negative intensity levels, several keyword groups were identified for each state. Except for the states of Michigan and Washington, the top topics identified were the same for both methods. Although we briefly examined lower-engagement topics as well, the number of associated comments was too limited to support meaningful conclusions; thus, we focused on the top topic for each state. Table 4 lists, for each state, the top topic descriptions—identified through manual review—along with a representative user comment.

Analysis showed that the top negatively charged topics across states were consistently generated by BERTopic rather than LDA. In California, for instance, all 8 leading topics were identified by BERTopic (Table 5).

**Table 4:** Summary of the most frequently occurring discussion topic in each of 11 US state-specific Reddit communities (California, Colorado, Iowa, Michigan, Minnesota, Ohio, Pennsylvania, Texas, Utah, Washington, and Wisconsin) from February 2022 to July 2024. Topic description was generated via manual review of combined BERTopic and latent Dirichlet allocation clusters, with an example user comment illustrating the theme.

State	Topic description	Example comment
California	Millions of birds, including over 50 million chickens nationwide and 5.1 million in 1 Iowa farm, have been culled due to a severe avian flu outbreak that is now causing significant shortages and supply chain disruptions in poultry and egg production. Comments also highlight that inhumane conditions in factory farms may be accelerating the virus’s spread, while ethically raised free-range chickens face higher exposure risks from wild birds.	“The Avian Flu has been going around and is really hitting NorCal hard right now. Tons of birds are having to be culled, reducing the stock and thus making good quality chicken scarcer.”
Colorado	Mass culling from avian flu outbreaks—citing figures like over 50 million birds nationwide and 85% of Colorado’s hens—has led to severe egg shortages and skyrocketing prices, with some cases noting price hikes from US $18 to US $97 per case. The debate also centers on whether Colorado’s new mandate requiring cage-free eggs is merely coincidental or exacerbating the crisis, as consumers rush to stock up, further straining supply chains.	“It’s the lack of regulation that exacerbated that avian bird flu situation. The fact that the cage free law (in CO) took effect in the new year is merely correlation, not causation. The egg crisis started months before the new CO law took effect and egg prices are up over 50% nationwide, including states with no such laws.”
Iowa	Dead birds being reported, canceled exhibits, and even personal experiences with “influenza A” are repeatedly cited as evidence that an avian flu outbreak is actively impacting local bird populations, including enforced culling and reduced displays at parks and fairs. Yet, while many express genuine concern over these effects—even linking H5N1 and ongoing pandemic pressures—others dismiss the phenomenon as a political hoax or overblown, underscoring a tension between alarm and skepticism.	“It’s not necessarily the cold that is killing them, birds tend to be very resilient to the cold, it is more likely bird flu or some other disease.”
Michigan	Egg prices are soaring, with some comments pointing to a supply shortage caused by avian flu—millions of egg-laying hens culled—and others decrying corporate greed that inflated profits by as much as 960% even when major producers avoided the outbreak. Many also debate whether factors like seasonal demand, feed and transportation costs, and industry concentration are exacerbating price hikes, with some sarcastically suggesting that companies might even manipulate flu outbreaks for profit.	“Honestly this food is ridiculous and I forget which subreddit it was but people posting egg prices tells you a lot and I know there were issues with the avian flu, but holy hell 8 bucks for a normal carton of 12 white eggs? No thank you mam. Also, I am semi worried this weather throughout California’s inlands may actually hurt the crops and cause even more increases to food across the country.”
Minnesota	The comments express concern over the bird flu outbreak, with several suggesting that infected birds—especially in farm settings—should be culled, and others remarking humorously about media coverage (like the “drunk birds” narrative) and comparing bird flu to other viruses. There is also debate over its potential to spread to humans, with some dismissing concerns, while others question the best methods to control the outbreak in commercial operations.	“They are likely suffering from this bird flu outbreak. You need to have them culled.”
Ohio	Comments focus on bird flu, with several users highlighting its threat—warning to mask up, avoid contact, and note its high pathogenicity—while others mix it with inflation, COVID-19, and even monkeypox. Opinions range from genuine alarm (“Bird Flu. Gonna die.”) to sarcastic dismissal (“They made up bird flu to disguise that they’re phasing out old surveillance technology.”).	“Covid is so over. Bird Flu! Is the hot new scary disease that you NEED to be CONCERNED about!”
Pennsylvania	Comments highlight that American factory farming practices—such as neglecting proper barn cleaning and recycling waste as cattle feed—contribute to the spread of avian flu and other health issues, contrasting sharply with European methods. They also note that massive culling of chickens (up to 49 million in some cases) has led to drastic reductions in egg production and soaring prices, with corporate price gouging and supply chain pressures further exacerbating the problem.	“We as a country allow the practice. How do you think the cows are getting bird flu? Some agriculture practices are better than others. Not everyone farms to the lowest common denominator, but some do. It’s not best practice to regrind old meat with fresh meat and sell it as hamburger, but it got done a lot until people started dying of e.coli and HUS syndrome.”
Texas	Egg prices have surged primarily because an avian flu outbreak forced producers to cull millions of chickens, drastically reducing the supply—cheaper factory-farm eggs jumped from about US $2 to US $5 per dozen, while free-range brands like Vital Farms maintained higher but stable prices. Commenters emphasize that inflation plays only a minor role compared to corporate profit-taking, price gouging, and differing farming practices that magnify the cost increase in poultry products.	“Eggs are not so expensive because of inflation. They are expensive because of bird flu making producers cull millions of chickens this year.”
Utah	Comments warn that avian flu is a serious health hazard, advising strict hygiene—like washing hands and cleaning clothing—to avoid picking up dead birds, as the virus can spread to other animals and (rarely) humans under high exposure. They reference recent reports from places like Antelope Island and link to articles and podcasts that detail dead birds testing positive and stress the potential risk if the virus jumps to humans.	“No advice about the duck but please wash your hands and be very very careful about handling, bird flu is serious.”
Washington	Egg prices have skyrocketed because a massive avian flu outbreak forced farmers to cull millions of egg-laying chickens, sharply reducing supply. Rising feed and energy costs, along with corporate profit-taking and supply chain disruptions, have compounded these effects while meat chickens remain less impacted.	“Multiple on multiple bird flu out breaks, combined with COVID screwing up supply chain issues. Both occuring so close together are hurting chicken and egg prices for both raw and prepared products.”, “I’m guessing bird flu may have had a component to this. Compound the loss of cheap labor and voila- expensive chicken.”
Wisconsin	Egg prices have surged primarily because the avian flu outbreak forced the culling of millions of egg-laying chickens, sharply reducing supply and triggering significant price hikes. While factors like rising feed and energy costs play a role, most commenters agree that the drastic loss of laying flocks—and subsequent corporate price gouging—is the main culprit driving up egg costs.	“Oh definitely. The main driver of egg prices right now is the bird flu killing millions of egg laying chickens. The chickens we eat for meat are divergent enough they are not impacted by this strain but yeah farmers are culling entire flocks because of this.”

**Table 5:** Comparison of the top discussion topic frequencies in California subreddit comments generated by BERTopic versus latent Dirichlet allocation (LDA) from February 2022 to July 2024, highlighting that contextual embeddings capture economic and biosecurity themes more comprehensively.

Topic	Frequency in comments, n (%)
BERTopic topic 4	25 (6.6)
BERTopic topic 3	25 (6.6)
BERTopic topic 5	25 (6.6)
BERTopic topic 2	25 (6.6)
BERTopic topic 1	25 (6.6)
BERTopic topic 6	18 (4.7)
BERTopic topic 8	12 (3.2)
BERTopic topic 7	12 (3.2)
LDA topic 8	11 (2.9)
BERTopic topic 9	11 (2.9)
BERTopic topic 10	10 (2.6)
LDA topic 1	10 (2.6)
LDA topic 22	10 (2.6)
LDA topic 11	9 (2.4)
LDA topic 4	9 (2.4)
BERTopic topic 11	8 (2.1)
LDA topic 2	8 (2.1)
BERTopic topic 12	7 (1.8)
BERTopic topic 13	7 (1.8)
LDA topic 3	7 (1.8)
BERTopic topic 14	6 (1.6)
LDA topic 27	6 (1.6)
LDA topic 19	5 (1.3)
LDA topic 12	5 (1.3)
LDA topic 20	5 (1.3)
LDA topic 10	5 (1.3)
LDA topic 9	5 (1.3)
LDA topic 18	5 (1.3)
LDA topic 16	4 (1.1)
LDA topic 21	4 (1.1)
LDA topic 7	4 (1.1)
LDA topic 6	4 (1.1)
LDA topic 13	4 (1.1)
LDA topic 30	3 (0.8)
LDA topic 26	3 (0.8)
LDA topic 25	3 (0.8)
LDA topic 28	3 (0.8)
LDA topic 14	3 (0.8)
LDA topic 5	3 (0.8)
LDA topic 24	2 (0.5)
LDA topic 15	1 (0.3)
LDA topic 17	1 (0.3)
LDA topic 29	1 (0.3)

## Discussion

### Overview

This work set out (1) to characterize the emotional trajectory of Reddit discourse during the 2022-2024 US H5N1 outbreak, (2) to identify the dominant discussion topics—especially those paired with negative sentiments, and (3) to examine whether those emotions and topics correlated with epidemiological severity at the national and state levels. Three primary findings emerged. First, negative emotions (sadness: n=901, 41.9%; anger: n=645, 30%; and fear: n=385, 17.9%) dominated conversation, fulfilling objective 1. Second, topic modeling revealed that economic narratives (mass culling causing egg shortages, leading to price spikes) best accounted for the highest‐intensity negative comments, achieving objective 2. Third, fear weakly tracked case counts in real time (*r*=0.11) while there were minimal correlations between the other sentiments and outbreak severity levels. However, as will be explored in more detail in the following discussion, further analysis revealed some nuanced patterns in how emotions align with outbreak trends—especially after accounting for temporal lags and outlier periods.

### Sentiments Versus Outbreaks Correlation Analysis

When further analyzing the sentiment intensity levels versus outbreak severity levels graphs, some interesting observations were made. Firstly, when examining the sentiment of “fear,” there was a minor positive correlation between “fear” intensity levels and outbreak severity levels, with a PCC of 0.11. This indicates a weak, positive relationship between the number of Reddit comments expressing fear and the weekly number of reported H5N1 cases. However, this weak correlation was not mirrored in the other emotions analyzed, such as “anger,” “sadness,” or “joy.”

A particularly interesting finding emerged when examining “anger,” “sadness,” and “joy” graphs in more detail: there appeared to be a temporal lag between outbreak severity and the intensity of these sentiments. To investigate this hypothesis further, the data points for the sentiment levels were shifted back by various numbers of weeks to account for the temporal delay. Significant findings resulted when shifting the data points back by 3 weeks; the PCC for the “anger,” “sadness,” and “joy” graphs increased from –0.019 to 0.204, 0.024 to 0.185, and 0.028 to 0.242, respectively, indicating weak positive correlations (Table 6). Furthermore, the overall sentiment levels’ correlation with the outbreak levels was also strengthened, with the PCC increasing from 0.022 to 0.203 (Figure 7A). Other shifts in data points, however, did not yield any significant results. On the other hand, the PCC for the “fear” graph only increased from 0.11 to 0.123, after applying the 3-week shift, which indicates that the sentiment of “fear” was not influenced by this temporal delay (Table 6).

**Table 6:** Pearson correlation coefficients (*r*) between weekly sentiment intensity scores (anger, fear, sadness, joy, and overall) and normalized H5N1 outbreak severity (0-100) across 11 US states from February 2022 to July 2024, comparing immediate associations, 3-week lag adjustments, January 2023 outlier removal, and combined adjustments.

Sentiment	Pearson *r* (immediate)	Pearson *r* (adjusted for 3-week delay)	Pearson *r* (adjusted for outlier month)	Pearson *r* (adjusted for 3-week delay and outlier month)
Fear	0.11	0.123	0.153	0.117
Anger	–0.019	0.204	0.084	0.245
Sadness	0.024	0.185	0.144	0.186
Joy	0.028	0.242	0.054	0.287
Overall	0.022	0.203	0.132	0.223

The delay could be attributed to several factors, including the time it takes for media coverage to accurately report on the severity of outbreaks, the gradual public realization of the true impact, or the time needed for information to spread widely and be absorbed. The notion of a lagged response is particularly relevant in modern times, where the flow of information may not always keep pace with events, especially during crises like outbreaks. The reason why the “fear” sentiment is not influenced by this phenomenon could be attributed to how it is our instinctive reaction to any negative situation, like an outbreak. In contrast, emotions like anger or sadness develop slower and may be influenced by broader factors, such as frustration over government responses or economic hardships due to the outbreak, which take time to manifest. Joy, too, may take a while to emerge, as the public awaits positive developments like successful management strategies or signs of recovery.

Another interesting observation is that “joy” levels, when shifted back by 3 weeks, displayed a PCC of 0.242 with the outbreak levels, which is now stronger than all other categories. This is an unexpected result because it contrasts the general consensus among past studies and society overall that outbreak severity should correspond inversely with public levels of joy. This finding could indicate that as the public becomes more aware of the outbreak’s severity, their response includes moments of relief or positive sentiment, possibly due to successful mitigation efforts, recovery reports, or effective public health measures that instill hope. Media coverage of such positive developments might take time to reach the public, aligning the increase in joy levels with prior outbreak levels.

However, this could also reflect another aspect of human nature where some individuals use humor or sarcasm in challenging situations like this pandemic as a coping mechanism. This phenomenon was also observed among several comments labeled as “joy.” Together, these findings provide a possible conclusion that public emotional reactions, within the context of this study, are not immediate, but rather unfold over time.

Another important observation that can be made is the significant peak in the overall and individual sentiment levels in January 2023, as shown in Figures 5 and 6A-D. This could be due to significant activity on Reddit during that month in comparison to the other months in the time frame of this study, which could have been caused by a significant news event, comments that sparked interest among a large number of people or caused long discussions, or purely a coincidence. In terms of correlation analysis, this significant deviation from the overall trend suggested that their inclusion might have overemphasized short-term social media engagement unrelated to outbreaks.

To isolate the impact of the data points in January 2023, they were removed from the dataset, and the correlation coefficients were recalculated. The results revealed that, following the removal of the outliers, the correlation between overall sentiment intensity and outbreak severity strengthened to 0.132 (Figure 7B). Additionally, the correlations for the rest of the sentiment level graphs were also improved, with the PCC for the “fear,” “anger,” “sadness,” and “joy” graphs increasing from 0.11 to 0.153, –0.019 to 0.084, 0.024 to 0.144, and 0.028 to 0.054, respectively (Table 6). Although these increases, as a whole, were not nearly as significant as the impacts of the 3-week shift, they were still enough to represent weak positive correlations between the sentiment intensity levels and outbreak severity levels.

This increase in correlation values indicates that the January 2023 peak was indeed an outlier that caused the insignificant PCCs calculated in the Results section. By eliminating the outlier’s influence, the analysis provides a more representative understanding of how public sentiment typically responds to changes in outbreak severity. The strengthened correlation now suggests a weak positive association between these 2 variables, a finding previously obscured by the exceptional circumstances of January 2023.

When both adjustments are applied—shifting sentiment scores 3 weeks backward and removing the January 2023 outliers—the associations tighten even more. The PCC values for the “fear,” “anger,” “sadness,” and “joy” graphs are now 0.117, 0.245, 0.186, and 0.287, respectively, with the overall PCC increasing to 0.223 (Table 6). These results suggest that, within the context of this study, once reporting delays and anomalous surges are accounted for, public emotions on Reddit tend to mirror epidemiological trends far more closely than raw, unadjusted data imply—though the relationships remain only moderate, reinforcing that sentiment is just one of several factors shaping web-based reactions to this outbreak. Figure 7C visualizes this convergence.

Figure 7D illustrates the original sentiment intensity levels alongside 3 adjusted sentiment intensity trend lines and the outbreak severity levels. Visual inspection clearly indicates a stronger correlation between the adjusted sentiment intensity trends and the outbreak severity levels compared to the original, unadjusted trend.

### Topic Modeling Analysis

When comparing the results of the BERTopic and LDA models, not much can be concluded by simply examining the most frequently appearing words in topics generated by both models, as there is too much detail and variation across individual states. However, by analyzing the tables produced for the most frequently appearing topics by state and topics with corresponding comments that exhibit the highest comprehensive negative intensity levels by state, it was observed that all of the top topics for both tables were generated by the BERTopic model instead of the LDA model. For example, Table 5 illustrates the identified topics ranked by the number of comments they correspond to for the state of California, and the top 8 topics were all generated by the BERTopic model. Similar trends were observed for other states’ comments. This finding suggests that, in the context of this study, the BERTopic model was much more effective than the commonly used LDA model at identifying the most frequently appearing topics and topics that are the most representative of comments showing negative emotions in the data. Based on the topics identified, the predominant concerns shared across all states are on the cyclical nature of the avian flu outbreak, the deaths of millions of birds, and the outbreak’s direct impact on egg and poultry supply, which in turn drives up prices dramatically. Commenters note that government-mandated culling has sharply reduced supply, and while factors like feed costs, transportation, and even new regulatory measures (such as cage-free mandates) play a role, many see corporate profiteering as a critical exacerbator of the crisis. In states such as California, Colorado, and Texas, there is a strong emphasis on the sheer scale of the culling and its visible effects on local poultry availability. Meanwhile, in Michigan, users debate whether high egg prices stem mainly from the outbreak or if additional economic factors like corporate greed are also at work. Other regions, such as Pennsylvania and Wisconsin, discuss how inhumane factory farming practices may accelerate the spread of the virus while also noting that the disruption of poultry supply chains contributes to broader food security concerns (Table 4).

The most prominent topic seems to be the increase in egg prices, with 6 of the 11 states’ comments having it as their most frequently discussed topic. In February 2022, egg prices averaged US $1.36 per dozen but surged over 100% to US $2.74 by July 2024, peaking at US $5.29 in December 2022 [20]. This price hike was driven by the recurring deaths of chickens, which are the species of poultry discussed the most in these Reddit comments. They have been particularly affected by the H5N1 avian influenza pandemic due to their high susceptibility to the virus and how they are mostly raised in densely packed environments. The high amount of deaths in chickens is not only due to the high mortality rates of the virus itself but also the mandatory culling policies instilled by the government, which required entire flocks of poultry to be depopulated if any cases of the disease emerged among the flock. For instance, in April 2024, Cal-Maine Foods, the largest US egg producer, had to cull 1.6 million hens in Texas following an outbreak. These measures, while crucial for containment, exacerbate the supply crisis and impact consumer costs [21]. Moreover, the economic strain extends beyond just egg prices. Farmers also faced significant financial losses due to the forced depopulation of their flocks, which led to broader concerns about food security and the long-term viability of poultry farming when facing recurrent outbreaks.

To identify less frequently discussed, yet important, topics, comments from each state and their associated topics were manually examined. Through manual analysis of contextual information derived from the BERTopic and LDA models, several additional themes were identified that, while not among the most dominant, appeared consistently across multiple states. These include (1) human transmission risks, highlighting fears around the virus potentially being a threat to humans and pets; (2) comparisons to past pandemics, with people drawing parallels to diseases such as COVID-19, swine flu, and severe acute respiratory syndrome; and (3) contamination prevention, including concerns over raw milk safety, pasteurization, and the closure of bird exhibits in public venues.

When examining the sentiment distributions of comments corresponding to each state’s number-one topic, a clear pattern emerges where negative emotions—primarily sadness and anger—dominate (Multimedia Appendix 1). Specifically, the topic evoking the highest level of sadness was Minnesota’s leading concern about the spread of avian influenza and related culling practices, with 66.7% (n=10) of comments expressing sadness. Texas’s top topic, centered around the dramatic rise in egg prices, provoked the most anger, evident in 68% (n=17) of the comments. Ohio’s primary topic regarding public warnings about a new flu strain generated the most fear among commenters, with 29.4% (n=5) of comments expressing fear. California’s dominant topic, consisting mainly of news updates and statistics regarding the bird flu, elicited universally negative sentiment, where 100% (n=25) of comments demonstrated a combination of sadness, anger, or fear. On the other hand, Utah’s top topic, focusing on the threat of the bird flu, the potential risk of it spreading to humans, and how we can prevent that from occurring, displayed the highest percentage of joyful comments, with 18.2% (n=2) of the comments labeled with joy (Multimedia Appendix 1).

Overall, based on the most prominent topics exhibited by these Reddit comments, people seem to be concerned about the outbreak’s cycle of infection, deaths of birds, culling, and rising prices, and how we could potentially balance between disease containment and economic sustainability.

### Comparison to Prior Work

The findings of this study align with a substantial body of literature examining public sentiment during infectious disease outbreaks via social media. Chew and Eysenbach [22], in their work on the 2009 H1N1 pandemic, similarly observed that fear and sadness dominated public sentiment on Twitter during the early stages of the outbreak. Ahmed et al [23] also found a weak or delayed correlation between case numbers and web-based sentiment, consistent with this study’s finding that public concern often lags behind epidemiological developments. The prominence of economic anxieties, such as concerns over egg prices in shaping negative sentiment, echoes the observations of Stieglitz et al [24], who noted that economic and political factors often dominate web-based discourse in crisis settings. This study’s use of BERTopic over LDA for thematic modeling is supported by Jelodar et al [25], who argued that more advanced topic modeling techniques are better suited for capturing subtle semantic nuances in health-related discussions. Moreover, the platform-specific insights drawn from Reddit reflect the conclusion of Suhail et al [26] that Reddit fosters unique, in-depth discourse around health topics compared to Twitter. The complex expression of joy, often through sarcasm or memes, mirrors the findings of Wicke and Bolognesi [27], who highlighted humor as a coping mechanism during the COVID-19 pandemic.

However, not all prior research aligns with these findings. Discrepancies may stem from differences in platform culture (Reddit vs Twitter), timing, or public perception of disease severity. For example, Abd-Alrazaq et al [28], in their analysis of early COVID-19 tweets, found a strong, near-immediate alignment between case surges and increases in public anxiety and negative sentiment, which contrasts with this study’s observation of sentiment lag. Similarly, Lwin et al [29] observed clear peaks in fear, anger, and sadness that corresponded directly with major epidemiological events, suggesting a more reactive emotional landscape than was evident in Reddit posts analyzed here. Overall, this study both confirms and challenges aspects of prior work, offering new insights into the unique emotional and thematic patterns that emerge in Reddit discussions during zoonotic disease outbreaks.

### Limitations

The study has its limitations, both in data collection and data processing. When collecting data for the Reddit comments, the total number of comments scraped was only around 2100, which is rather low compared to some other similar studies, such as a 2016 study on avian influenza risk surveillance in North America collected 38,191 Twitter comments for their dataset [8]. This limited number of comments scraped was due to the manual scraping methods, which involved manually selecting a list of words and phrases that were deemed relevant to the H5N1 virus and manually identifying the subreddits based on geographical location names. As a result, certain relevant comments not containing the keywords and additional state-specific subreddits with nongeographical names may have been overlooked. In terms of data processing, during the sentiment classification process, the fine-tuned BERT model that was used had an accuracy of around 94.2% (n=74,196), which means that there were inaccuracies in labeling the Reddit comments. In addition, there could have been mistakes in the labeling of the dataset used to train the model, which could have caused further inaccuracies. By examining the comments and the labels, these inaccuracies were noticeable, especially among the comments labeled as exhibiting “joy.” Many of them did not exhibit joyful sentiment but instead contained superficially positive words. This might have also been the reason for the unexpectedly high correlation between joy intensity levels and outbreak severity levels after applying the 3-week shift, as discussed previously. Additionally, the topic modeling analysis, which involved manual examination of keywords generated by BERTopic and LDA models, may have introduced subjective biases that reduced the quality of the results.

These limitations suggest that the study’s findings should be interpreted with caution, as the small datasets, potential inaccuracies in sentiment classification, and subjective elements in topic modeling may have impacted the results. Future research could address these issues by using larger and more diverse datasets, leveraging automated and dynamic data collection methods, and systematically testing multiple advanced models to identify those with the highest accuracy and robustness for both sentiment analysis and topic modeling.

### Generalizability

While this study offers valuable insights into public sentiment and discourse surrounding the H5N1 outbreak, its generalizability is subject to certain limitations. The analysis is based solely on Reddit data, which may not fully represent the broader population, particularly older age groups or individuals less active on social media. Additionally, the study only focused on subreddits from US states, which may limit the applicability of findings to other regions or countries with different demographic, cultural, or epidemiological contexts. Nevertheless, Reddit’s diverse and decentralized community structure provides a unique lens into localized public responses, and the methods used here can be adapted to other social media platforms and geographies. Future research could enhance generalizability by incorporating multiplatform data and including a wider range of geographic regions.

### Practical Implications

The findings of this study have significant implications for public health communication and policymaking during health crises like H5N1 outbreaks. The predominance of negative emotions such as anger, sadness, and fear in social media discussions reflects widespread public concern and anxiety. By identifying these sentiments and their associated topics, public health authorities can tailor their communication strategies to address specific concerns, such as economic hardships caused by rising egg prices or fears about the virus spreading among bird populations. Messaging that directly addresses these concerns can help alleviate public anxiety and foster trust in health authorities. Regional variations in sentiment and topic focus highlight the need for localized public health interventions. For example, states experiencing multiple outbreak spikes, such as Minnesota and Iowa, exhibited sustained negative sentiment levels, suggesting a need for ongoing communication efforts in these regions. Conversely, states with single major outbreak spikes might benefit from targeted campaigns addressing immediate concerns during the crisis. These insights can guide resource allocation and ensure that interventions are both timely and relevant to affected communities. Moreover, the use of social media analysis provides a dynamic tool for crisis management. Real-time monitoring of sentiment trends can help authorities predict public reactions to outbreak developments, enabling proactive responses. For instance, early detection of spikes in fear or anger could prompt immediate outreach efforts to mitigate panic and misinformation. This approach underscores the potential of social media as a complementary tool for traditional surveillance systems in public health.

### Future Directions

Future research should aim to overcome the limitations of this study by expanding the scope of data collection and analysis. Increasing the dataset size through automated and dynamic scraping methods across multiple social media platforms would provide a more comprehensive understanding of public sentiment. Platforms such as Twitter, Instagram, and Facebook, in addition to Reddit, could offer diverse perspectives and reduce biases inherent to any single platform’s user base. Advancements in analytical techniques could further enhance the robustness and granularity of findings. For example, integrating dynamic time warping methods for temporal analysis could better capture the delayed responses observed in this study. Similarly, using ensemble models for sentiment classification may improve accuracy and account for the nuanced emotional expressions found in social media comments. Comparative evaluations of these models could identify the most effective approaches for future studies. Real-time monitoring systems represent another promising avenue for development. By creating dashboards that integrate sentiment analysis with outbreak data, public health authorities could gain actionable insights to guide their responses. Such systems could be invaluable for adapting strategies in rapidly evolving crises, ensuring that interventions are both timely and effective. Cross-cultural studies offer additional opportunities to explore the generalizability of these findings. Applying similar analyses in different countries or cultural contexts could reveal whether observed patterns of public sentiment and reaction dynamics hold globally. These comparisons might also uncover unique challenges and opportunities for public health communication in diverse settings. Finally, interdisciplinary collaboration should be prioritized to leverage insights from fields such as epidemiology, behavioral science, and computational linguistics. Combining these perspectives could lead to more holistic public health strategies, integrating an understanding of disease dynamics with the social and emotional responses they provoke. By addressing these areas, future research can build on this study’s findings to improve public health preparedness and response during health crises.

### Conclusions

Overall, this study analyzed Reddit comments from 2022 to 2024 to assess public sentiments and topics related to the H5N1 avian influenza outbreak in the United States. By focusing on state-specific subreddits, it aimed to uncover prevalent emotions, key discussion topics, and correlations between public sentiment and outbreak severity. Despite limitations like a small text dataset and potential inaccuracies in sentiment classification, the study underscores social media’s value in gauging public sentiment during health crises. Understanding these sentiments can help public health authorities improve communication strategies and address public concerns.

## Appendix

Multimedia Appendix 1 State-level emotion distributions (anger, sadness, fear, and joy) for each state’s most prevalent BERTopic cluster, encompassing the 11 study states from February 2022 to July 2024 and revealing regional differences in affective responses.

## Abbreviations

API: application programming interface
BERT: Bidirectional Encoder Representations From Transformers
LDA: latent Dirichlet allocation
PCC: Pearson correlation coefficient
USDA: US Department of Agriculture

## Data Availability

Data are deposited in publicly available repositories [30].

## References

[1] (2024). Influenza: A(H5N1). World Health Organization.

[2] (2024). H5 bird flu: current situation. Centers for Disease Control and Prevention.

[3] (2024). Highly pathogenic avian influenza—RT-PCR diagnostics. Thermo Fisher Scientific.

[4] Cooper L, Diaz MI, Hanna JJ, Most ZM, Lehmann CU, Medford RJ (2025). Birds of a feather? Mis- and dis-information on the social media platform X related to avian influenza. Antimicrob Steward Healthc Epidemiol.

[5] Munaf S, Swingler K, Brülisauer Franz, O'Hare A, Gunn G, Reeves A (2023). Spatio-temporal evaluation of social media as a tool for livestock disease surveillance. One Health.

[6] Yousefinaghani S, Dara R, Poljak Z, Bernardo TM, Sharif S (2019). The assessment of Twitter's potential for outbreak detection: avian influenza case study. Sci Rep.

[7] McClaughlin E, Elliott S, Jewitt S, Smallman-Raynor M, Dunham S, Parnell T, Clark M, Tarlinton R (2024). UK flockdown: a survey of smallscale poultry keepers and their understanding of governmental guidance on highly pathogenic avian influenza (HPAI). Prev Vet Med.

[8] Robertson C, Yee L (2016). Avian influenza risk surveillance in North America with online media. PLoS One.

[9] (2024). Confirmations of highly pathogenic avian influenza in commercial and backyard flocks. US Department of Agriculture.

[10] PullPush.

[11] BERT base model (uncased). Hugging Face.

[12] Nidula E (2024). Emotions. Kaggle.

[13] Grinberg M, Debbeler J BERTopic: neural topic modeling. BERTopic.

[14] Radim R (2024). LdaModel. GENSIM topic modeling for humans.

[15] Radim R CoherenceModel. GENSIM topic modeling for humans.

[16] (2012). Ethics. AOIR.

[17] (2024). Codes and guidelines. ESOMAR.

[18] Eysenbach G, Till J (2001). Ethical issues in qualitative research on internet communities. BMJ.

[19] Effortlessly show statistical significance on seaborn plots. GitConnected.

[20] (2024). Eggs US. Trading Economics.

[21] (2024). Bird flu outbreak is driving up egg prices—again. CBS News.

[22] Chew C, Eysenbach G (2010). Pandemics in the age of Twitter: content analysis of tweets during the 2009 H1N1 outbreak. PLoS One.

[23] Ahmed W, Bath PA, Sbaffi L, Demartini G (2019). Novel insights into views towards H1N1 during the 2009 pandemic: a thematic analysis of Twitter data. Health Info Libr J.

[24] Stieglitz S, Mirbabaie M, Ross B, Neuberger C (2018). Social media analytics—challenges in topic discovery, data collection, and data preparation. Int J Inf Manag.

[25] Jelodar H, Wang Y, Yuan C, Feng X, Jiang X, Li Y, Zhao L (2018). Latent Dirichlet allocation (LDA) and topic modeling: models, applications, a survey. Multimed Tools Appl.

[26] Suhail L, Masood S, Haider A (2024). A comparative study of sentiment analysis for mental health related posts at Reddit & Twitter using machine learning and pre-trained models. J Innov Comput Emerg Technol.

[27] Wicke P, Bolognesi M (2020). Framing COVID-19: how we conceptualize and discuss the pandemic on Twitter. PLoS One.

[28] Abd-Alrazaq A, Alhuwail D, Househ M, Hamdi M, Shah Z (2020). Top concerns of tweeters during the COVID-19 pandemic: infoveillance study. J Med Internet Res.

[29] Lwin M, Lu J, Sheldenkar A, Schulz P, Shin W, Gupta R, Yang Y (2020). Global sentiments surrounding the COVID-19 pandemic on Twitter: analysis of twitter trends. JMIR Public Health Surveill.

[30] Pang O (2024). H5N1 social media analysis. GitHub.

