# Supplementary figures and images for: Analyzing Reddit Social Media Content in the United States Related to H5N1: Sentiment and Topic Modeling Study
Source: J Med Internet Res. 2025 Sep 9;27:e70746. doi: 10.2196/70746 (PMC12457856; doi:10.2196/70746)

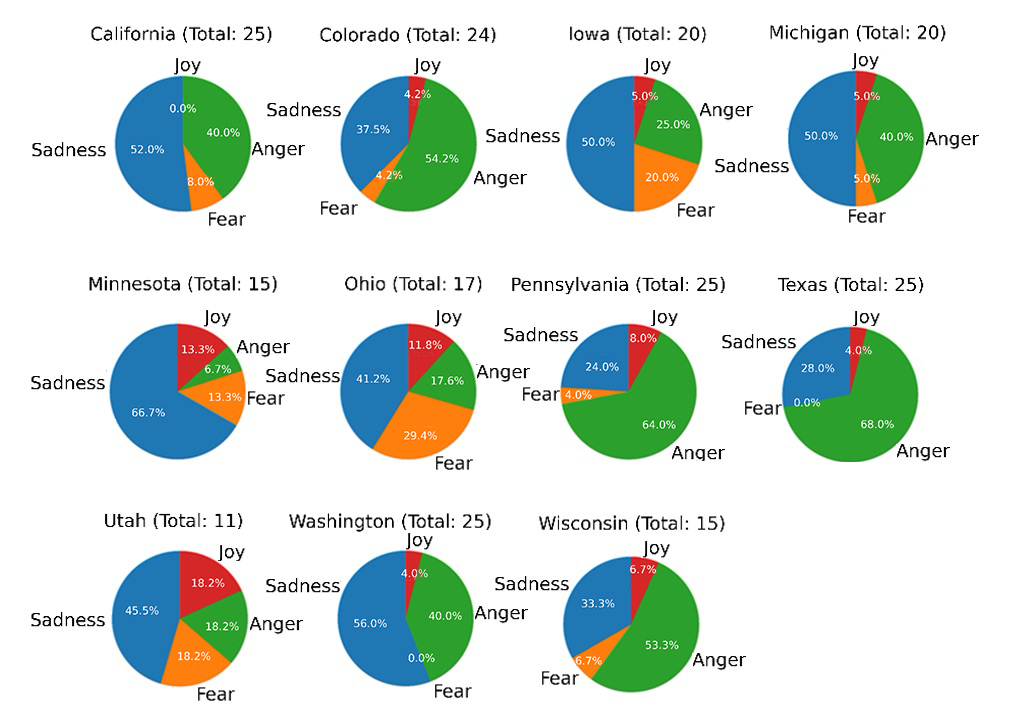

Supplement: Multimedia Appendix 1 [file jmir_v27i1e70746_app1.png]
